# Supplementary material for: Diverse winter communities and biogeochemical cycling potential in the under-ice microbial plankton of a subarctic river-to-sea continuum
Source: Microbiol Spectr. 2024 Mar 21;12(5):e04160-23. doi: 10.1128/spectrum.04160-23 (PMC11210273; doi:10.1128/spectrum.04160-23)
Supplement: Supplemental material — Supplemental figures and tables. [file spectrum.04160-23-s0001.docx]

**Supplementary Material**

Diverse winter communities and biogeochemical cycling potential in the under-ice microbial plankton of a subarctic river-to-sea continuum

Marie-Amélie Blais^a,b,c,d^#*, Warwick F. Vincent^a,b,c,d^, Adrien Vigneron^a,b,c,d^, Aurélie Labarre^a,b,d,e^, Alex Matveev^a,c,d^*, Lígia Fonseca Coelho^f,g,h^*, and Connie Lovejoy^a,b,d,e^

^a^Département de Biologie, Université Laval, Quebec City, QC, Canada

^b^Institut de Biologie Intégrative et des Systèmes (IBIS), Université Laval, Quebec City, QC, Canada

^c^Centre for Northern Studies (CEN), Université Laval, Quebec City, QC, Canada

^d^Takuvik Joint International Laboratory, Université Laval, Quebec City, QC, Canada

^e^Québec-Océan, Université Laval, Quebec City, QC, Canada

^f^Centro de Química Estrutural, Departamento de Engenharia Química, Instituto Superior Técnico, Universidade de Lisboa, Lisboa, Portugal

^g^Institute for Bioengineering and Biosciences, Instituto Superior Técnico, Universidade de Lisboa, Lisboa, Portugal

^h^Associate Laboratory i4HB—Institute for Health and Bioeconomy at Instituto Superior Técnico,

Universidade de Lisboa, Lisboa, Portugal

Running Head: winter microbiome of an ice-covered river

**Supplementary Figures**

Figure S1. Spearman correlation matrix of limnological variables.

Figure S2. Relative abundance of the 50 most relatively abundant prokaryotes taxa.

Figure S3. Relative abundance of the 50 most relatively abundant microbial eukaryotes.

Figure S4. Ward hierarchical clustering of the metagenome based on KOs for the coassembly and the reads.

Figure S5. Taxonomic classification of the contigs with genes encoding for *hao* at phylum level (a) and for *pmo-amo* at phylum (b) and order (c) level.

**Supplementary Tables**

Table S1. List of KO used for Figure 5.

Table S2. Differential abundance analysis results for the KOs listed in Table S1 that are significantly (*p*≤0.01) differentially abundant along the river between the shallow and the deeper sites (*RSh* vs. *R*).

Table S3. Differential abundance analysis results for the KOs listed in Table S1 that are significantly (*p*≤0.01) differentially abundant in the vertical plume profile between surface water and brackish water at 4 m depth (*PS* vs. *P4M*).

**
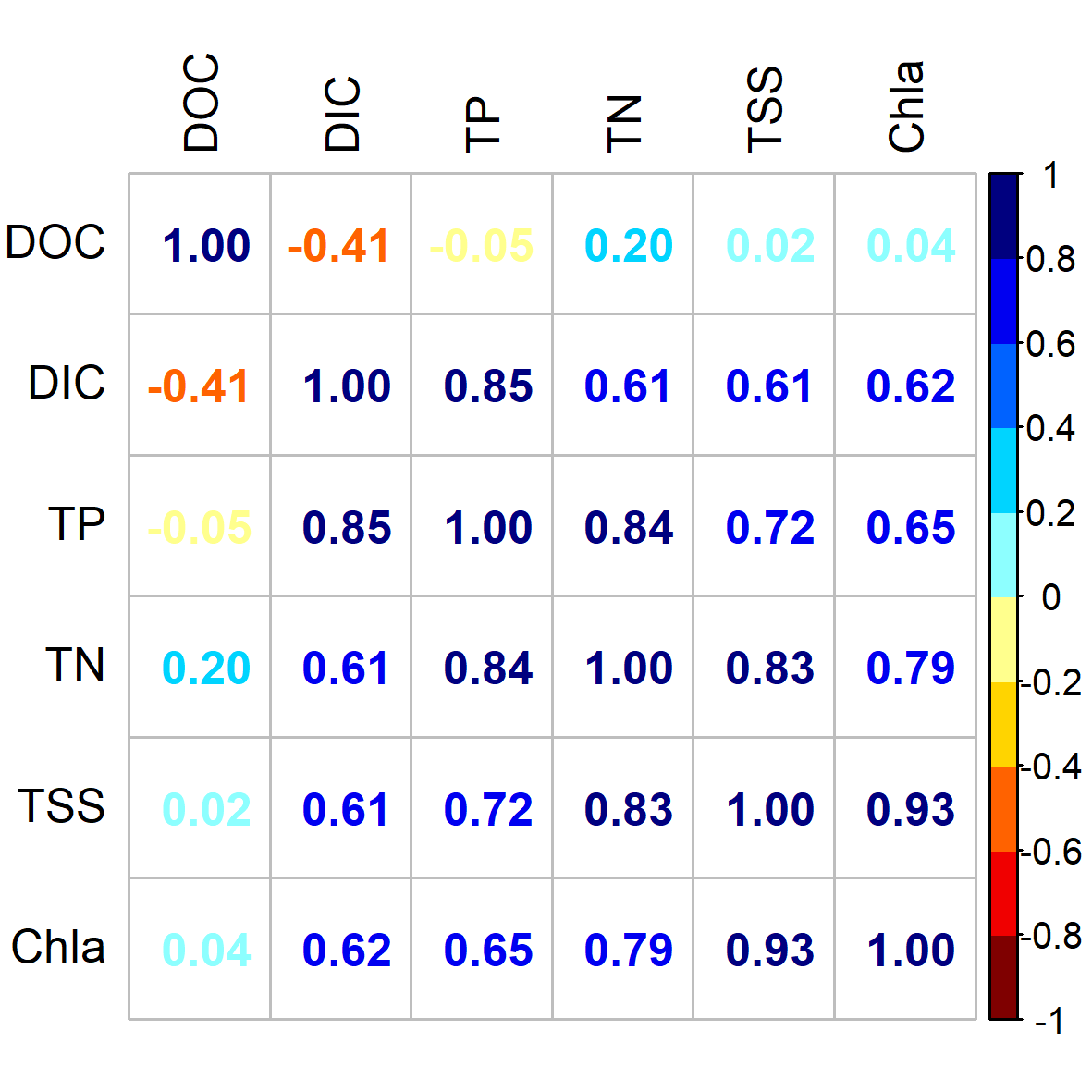
**

**Figure S1.** Spearman correlation matrix of limnological variables.


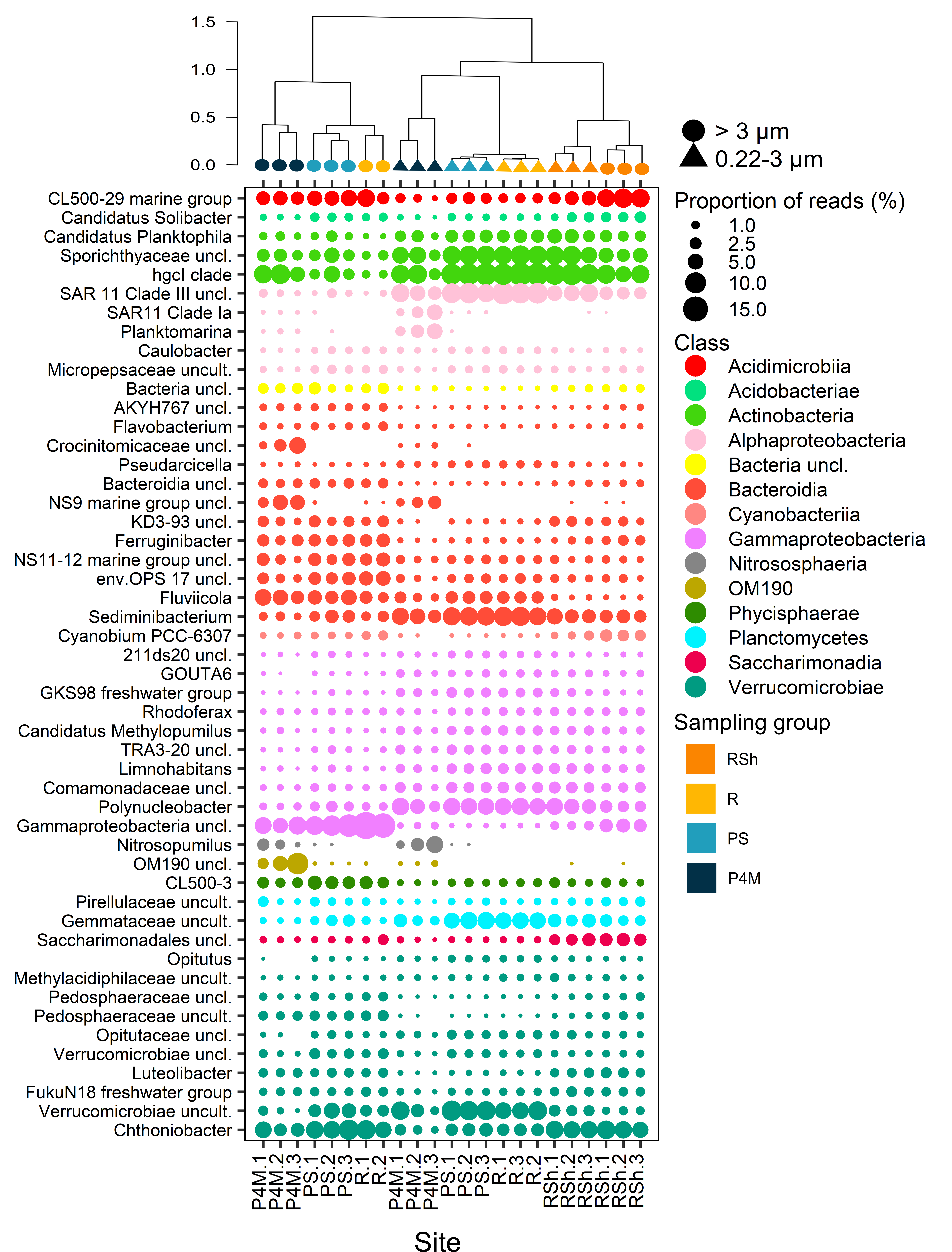


**Figure S2.** Proportion of reads (%) of the 50 most relatively abundant prokaryotes taxa (determined by the sum of relative abundance of all samples) aggregated to their highest taxonomic resolution. The color of the points correspond to the class and the size to the proportion of reads. Samples are ordered according to Ward hierarchical clustering calculated at the ASV level.


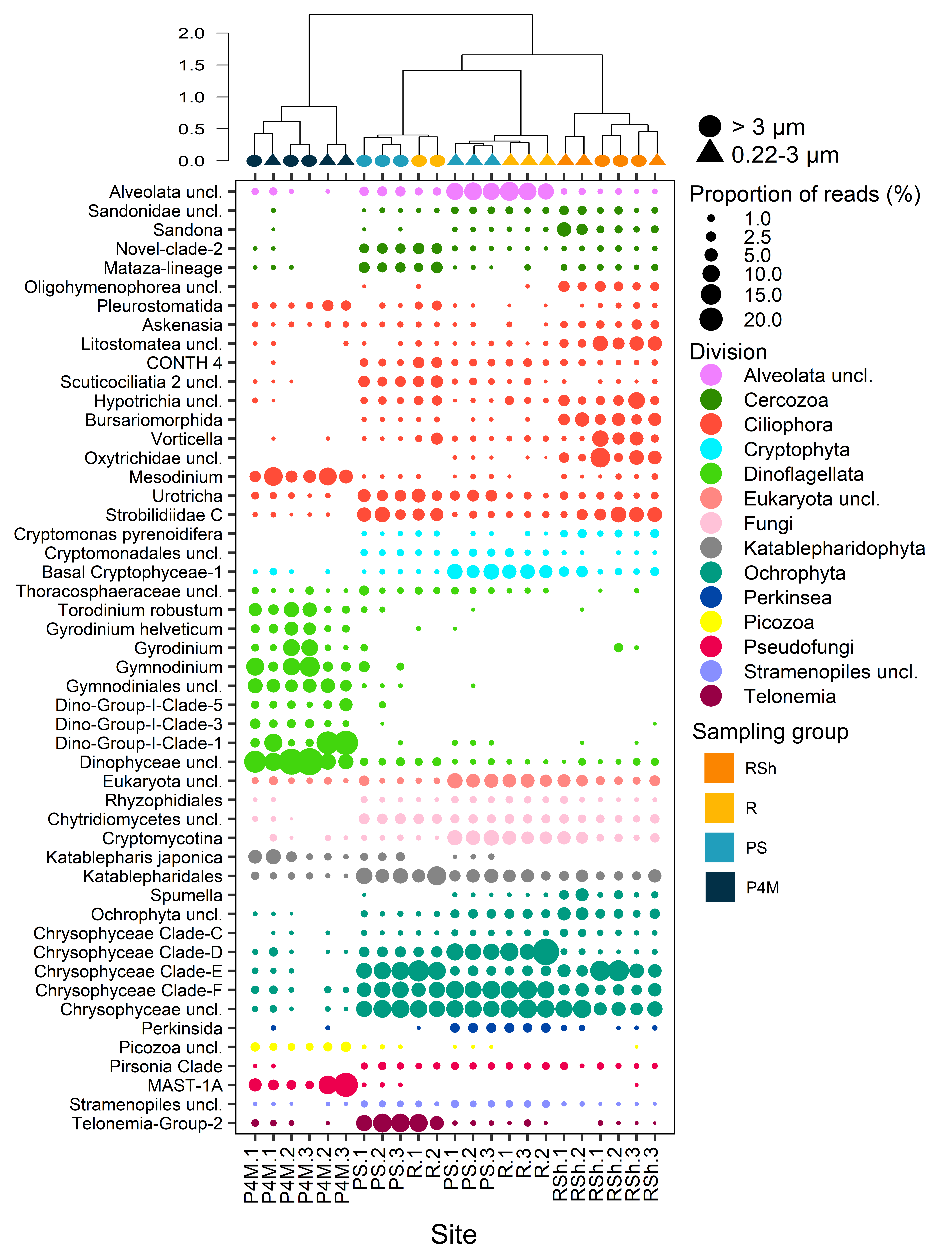


**Figure S3.** Proportion of reads (%) of the 50 most relatively abundant microbial eukaryotes taxa (determined by the sum of relative abundance of all samples) aggregated to their highest taxonomic resolution. The color of the points correspond to the division and the size to the proportion of reads. Samples are ordered according to Ward hierarchical clustering calculated at the ASV level.


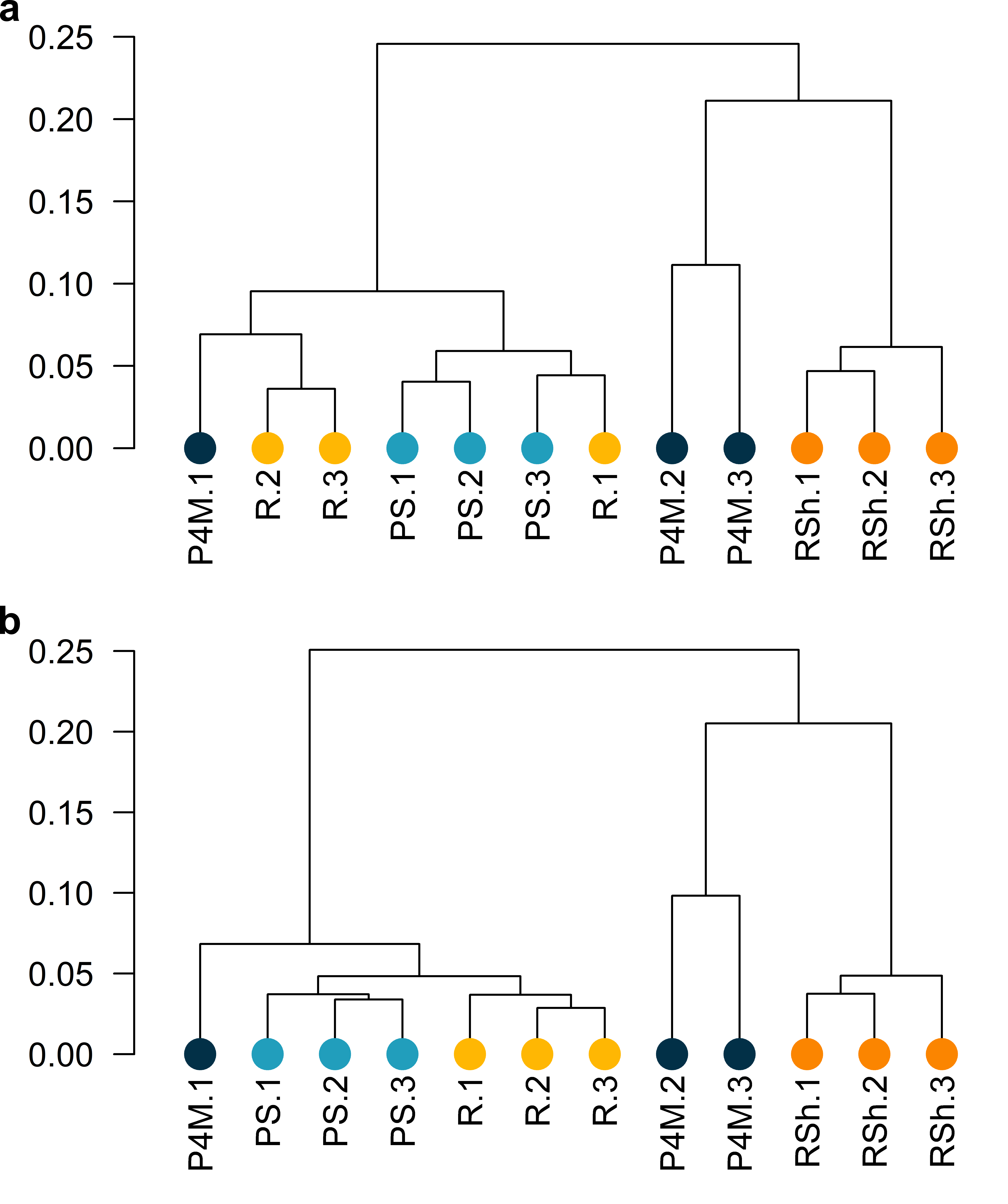


**Figure S4.** Ward hierarchical clustering of the metagenome based on KOs for **a**) the coassembly and **b**) the reads. Colors correspond to the sampling group as in Figure 3.1.


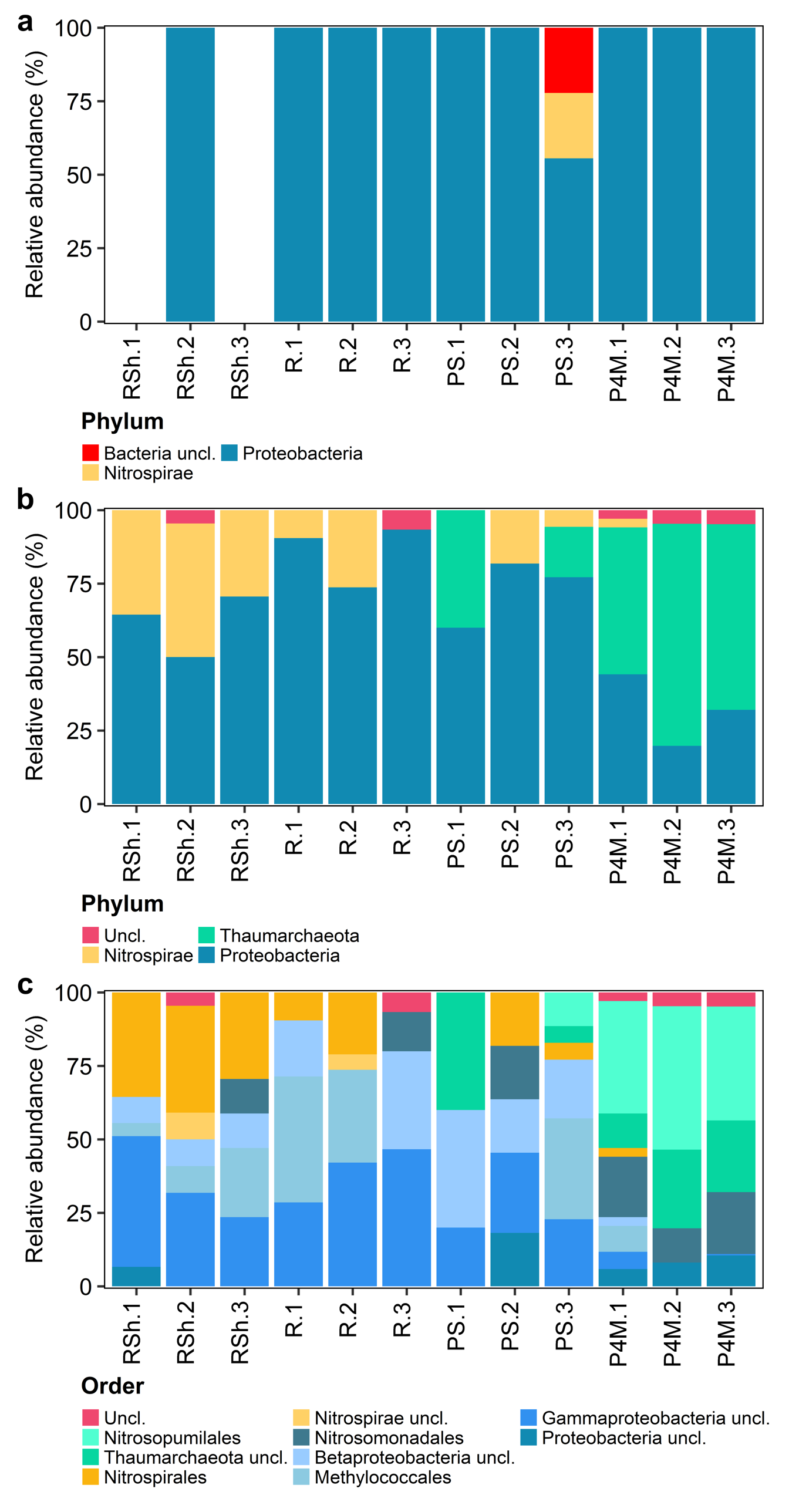


**Figure S5.** Taxonomic classification of the contigs with genes encoding for *hao* at phylum level (**a**) and for *pmo-amo* at phylum (**b**) and order (**c**) level.

**Table S1.** List of KO used for Figure 5.

| KO | Gene | Name | Pathway | Module or Reaction |
| --- | --- | --- | --- | --- |
| K01847 | *MUT* | methylmalonyl-CoA mutase [EC:5.4.99.2] | Carbon metabolism | 3-Hydroxypropionate bi-cycle |
| K01848 | *E5.4.99.2A* | methylmalonyl-CoA mutase, N-terminal domain [EC:5.4.99.2] | Carbon metabolism | 3-Hydroxypropionate bi-cycle |
| K01849 | *E5.4.99.2B* | methylmalonyl-CoA mutase, C-terminal domain [EC:5.4.99.2] | Carbon metabolism | 3-Hydroxypropionate bi-cycle |
| K01961 | *accC* | acetyl-CoA carboxylase, biotin carboxylase subunit [EC:6.4.1.2 6.3.4.14] | Carbon metabolism | 3-Hydroxypropionate bi-cycle |
| K01962 | *accA* | acetyl-CoA carboxylase carboxyl transferase subunit alpha [EC:6.4.1.2 2.1.3.15] | Carbon metabolism | 3-Hydroxypropionate bi-cycle |
| K01963 | *accD* | acetyl-CoA carboxylase carboxyl transferase subunit beta [EC:6.4.1.2 2.1.3.15] | Carbon metabolism | 3-Hydroxypropionate bi-cycle |
| K02160 | *accB* | acetyl-CoA carboxylase biotin carboxyl carrier protein | Carbon metabolism | 3-Hydroxypropionate bi-cycle |
| K05606 | *MCEE* | methylmalonyl-CoA/ethylmalonyl-CoA epimerase [EC:5.1.99.1] | Carbon metabolism | 3-Hydroxypropionate bi-cycle |
| K08691 | *mcl* | malyl-CoA/(S)-citramalyl-CoA lyase [EC:4.1.3.24 4.1.3.25] | Carbon metabolism | 3-Hydroxypropionate bi-cycle |
| K09709 | *meh* | 3-methylfumaryl-CoA hydratase [EC:4.2.1.153] | Carbon metabolism | 3-Hydroxypropionate bi-cycle |
| K14449 | *mch* | 2-methylfumaryl-CoA hydratase [EC:4.2.1.148] | Carbon metabolism | 3-Hydroxypropionate bi-cycle |
| K00031 | *IDH1* | isocitrate dehydrogenase [EC:1.1.1.42] | Carbon metabolism | Arnon-Buchanan cycle |
| K00174 | *korA* | 2-oxoglutarate/2-oxoacid ferredoxin oxidoreductase subunit alpha [EC:1.2.7.3 1.2.7.11] | Carbon metabolism | Arnon-Buchanan cycle |
| K00175 | *korB* | 2-oxoglutarate/2-oxoacid ferredoxin oxidoreductase subunit beta [EC:1.2.7.3 1.2.7.11] | Carbon metabolism | Arnon-Buchanan cycle |
| K00176 | *korD* | 2-oxoglutarate ferredoxin oxidoreductase subunit delta [EC:1.2.7.3] | Carbon metabolism | Arnon-Buchanan cycle |
| K00177 | *korC* | 2-oxoglutarate ferredoxin oxidoreductase subunit gamma [EC:1.2.7.3] | Carbon metabolism | Arnon-Buchanan cycle |
| K00242 | *sdhD* | succinate dehydrogenase / fumarate reductase, membrane anchor subunit | Carbon metabolism | Arnon-Buchanan cycle |
| K00244 | *frdA* | fumarate reductase flavoprotein subunit [EC:1.3.5.4] | Carbon metabolism | Arnon-Buchanan cycle |
| K00245 | *frdB* | fumarate reductase iron-sulfur subunit [EC:1.3.5.4] | Carbon metabolism | Arnon-Buchanan cycle |
| K00246 | *frdC* | fumarate reductase subunit C | Carbon metabolism | Arnon-Buchanan cycle |
| K00247 | *frdD* | fumarate reductase subunit D | Carbon metabolism | Arnon-Buchanan cycle |
| K01006 | *ppdK* | pyruvate, orthophosphate dikinase [EC:2.7.9.1] | Carbon metabolism | Arnon-Buchanan cycle |
| K01676 | *E4.2.1.2A* | fumarate hydratase, class I [EC:4.2.1.2] | Carbon metabolism | Arnon-Buchanan cycle |
| K01681 | *ACO* | aconitate hydratase [EC:4.2.1.3] | Carbon metabolism | Arnon-Buchanan cycle |
| K01682 | *acnB* | aconitate hydratase 2 / 2-methylisocitrate dehydratase [EC:4.2.1.3 4.2.1.99] | Carbon metabolism | Arnon-Buchanan cycle |
| K01958 | *PC* | pyruvate carboxylase [EC:6.4.1.1] | Carbon metabolism | Arnon-Buchanan cycle |
| K01959 | *pycA* | pyruvate carboxylase subunit A [EC:6.4.1.1] | Carbon metabolism | Arnon-Buchanan cycle |
| K01960 | *pycB* | pyruvate carboxylase subunit B [EC:6.4.1.1] | Carbon metabolism | Arnon-Buchanan cycle |
| K03737 | *por* | pyruvate-ferredoxin/flavodoxin oxidoreductase [EC:1.2.7.1 1.2.7.-] | Carbon metabolism | Arnon-Buchanan cycle |
| K15230 | *aclA* | ATP-citrate lyase alpha-subunit [EC:2.3.3.8] | Carbon metabolism | Arnon-Buchanan cycle |
| K15231 | *aclB* | ATP-citrate lyase beta-subunit [EC:2.3.3.8] | Carbon metabolism | Arnon-Buchanan cycle |
| K15232 | *ccsA* | citryl-CoA synthetase large subunit [EC:6.2.1.18] | Carbon metabolism | Arnon-Buchanan cycle |
| K15234 | *ccl* | citryl-CoA lyase [EC:4.1.3.34] | Carbon metabolism | Arnon-Buchanan cycle |
| K18556 | *frdA* | NADH-dependent fumarate reductase subunit A [EC:1.3.1.6] | Carbon metabolism | Arnon-Buchanan cycle |
| K18560 | *frdE* | NADH-dependent fumarate reductase subunit E | Carbon metabolism | Arnon-Buchanan cycle |
| K01679 | *E4.2.1.2B* | fumarate hydratase, class II [EC:4.2.1.2] | Carbon metabolism | Arnon-Buchanan cycle/3-Hydroxypropionate bi-cycle |
| K00239 | *sdhA* | succinate dehydrogenase / fumarate reductase, flavoprotein subunit [EC:1.3.5.1 1.3.5.4] | Carbon metabolism | Arnon-Buchanan cycle/3-Hydroxypropionate bi-cycle/Dicarboxylate-hydroxybutyrate cycle |
| K00240 | *sdhB* | succinate dehydrogenase / fumarate reductase, iron-sulfur subunit [EC:1.3.5.1 1.3.5.4] | Carbon metabolism | Arnon-Buchanan cycle/3-Hydroxypropionate bi-cycle/Dicarboxylate-hydroxybutyrate cycle |
| K00241 | *sdhC* | succinate dehydrogenase / fumarate reductase, cytochrome b subunit | Carbon metabolism | Arnon-Buchanan cycle/3-Hydroxypropionate bi-cycle/Dicarboxylate-hydroxybutyrate cycle |
| K00024 | *mdh* | malate dehydrogenase [EC:1.1.1.37] | Carbon metabolism | Arnon-Buchanan cycle/Dicarboxylate-hydroxybutyrate cycle |
| K00169 | *porA* | pyruvate ferredoxin oxidoreductase alpha subunit [EC:1.2.7.1] | Carbon metabolism | Arnon-Buchanan cycle/Dicarboxylate-hydroxybutyrate cycle |
| K00170 | *porB* | pyruvate ferredoxin oxidoreductase beta subunit [EC:1.2.7.1] | Carbon metabolism | Arnon-Buchanan cycle/Dicarboxylate-hydroxybutyrate cycle |
| K00171 | *porD* | pyruvate ferredoxin oxidoreductase delta subunit [EC:1.2.7.1] | Carbon metabolism | Arnon-Buchanan cycle/Dicarboxylate-hydroxybutyrate cycle |
| K00172 | *porC* | pyruvate ferredoxin oxidoreductase gamma subunit [EC:1.2.7.1] | Carbon metabolism | Arnon-Buchanan cycle/Dicarboxylate-hydroxybutyrate cycle |
| K01007 | *pps* | pyruvate, water dikinase [EC:2.7.9.2] | Carbon metabolism | Arnon-Buchanan cycle/Dicarboxylate-hydroxybutyrate cycle |
| K01595 | *ppc* | phosphoenolpyruvate carboxylase [EC:4.1.1.31] | Carbon metabolism | Arnon-Buchanan cycle/Dicarboxylate-hydroxybutyrate cycle |
| K01677 | *E4.2.1.2AA* | fumarate hydratase subunit alpha [EC:4.2.1.2] | Carbon metabolism | Arnon-Buchanan cycle/Dicarboxylate-hydroxybutyrate cycle |
| K01678 | *E4.2.1.2AB* | fumarate hydratase subunit beta [EC:4.2.1.2] | Carbon metabolism | Arnon-Buchanan cycle/Dicarboxylate-hydroxybutyrate cycle |
| K01902 | *sucD* | succinyl-CoA synthetase alpha subunit [EC:6.2.1.5] | Carbon metabolism | Arnon-Buchanan cycle/Dicarboxylate-hydroxybutyrate cycle |
| K01903 | *sucC* | succinyl-CoA synthetase beta subunit [EC:6.2.1.5] | Carbon metabolism | Arnon-Buchanan cycle/Dicarboxylate-hydroxybutyrate cycle |
| K00134 | *GAPDH* | glyceraldehyde 3-phosphate dehydrogenase [EC:1.2.1.12] | Carbon metabolism | Calvin cycle |
| K00150 | *gap2* | glyceraldehyde-3-phosphate dehydrogenase (NAD(P)) [EC:1.2.1.59] | Carbon metabolism | Calvin cycle |
| K00615 | *E2.2.1.1* | transketolase [EC:2.2.1.1] | Carbon metabolism | Calvin cycle |
| K00855 | *PRK* | phosphoribulokinase [EC:2.7.1.19] | Carbon metabolism | Calvin cycle |
| K00927 | *PGK* | phosphoglycerate kinase [EC:2.7.2.3] | Carbon metabolism | Calvin cycle |
| K01086 | *fbp-SEBP* | fructose-1,6-bisphosphatase I / sedoheptulose-1,7-bisphosphatase [EC:3.1.3.11 3.1.3.37] | Carbon metabolism | Calvin cycle |
| K01100 | *E3.1.3.37* | sedoheptulose-bisphosphatase [EC:3.1.3.37] | Carbon metabolism | Calvin cycle |
| K01601 | *rbcL* | ribulose-bisphosphate carboxylase large chain [EC:4.1.1.39] | Carbon metabolism | Calvin cycle |
| K01602 | *rbcS* | ribulose-bisphosphate carboxylase small chain [EC:4.1.1.39] | Carbon metabolism | Calvin cycle |
| K01623 | *ALDO* | fructose-bisphosphate aldolase, class I [EC:4.1.2.13] | Carbon metabolism | Calvin cycle |
| K01624 | *FBA* | fructose-bisphosphate aldolase, class II [EC:4.1.2.13] | Carbon metabolism | Calvin cycle |
| K01807 | *rpiA* | ribose 5-phosphate isomerase A [EC:5.3.1.6] | Carbon metabolism | Calvin cycle |
| K01808 | *rpiB* | ribose 5-phosphate isomerase B [EC:5.3.1.6] | Carbon metabolism | Calvin cycle |
| K02446 | *glpX* | fructose-1,6-bisphosphatase II [EC:3.1.3.11] | Carbon metabolism | Calvin cycle |
| K03841 | *FBP* | fructose-1,6-bisphosphatase I [EC:3.1.3.11] | Carbon metabolism | Calvin cycle |
| K05298 | *GAPA* | glyceraldehyde-3-phosphate dehydrogenase (NADP+) (phosphorylating) [EC:1.2.1.13] | Carbon metabolism | Calvin cycle |
| K11532 | *glpX-SEBP* | fructose-1,6-bisphosphatase II / sedoheptulose-1,7-bisphosphatase [EC:3.1.3.11 3.1.3.37] | Carbon metabolism | Calvin cycle |
| K00626 | *ACAT* | acetyl-CoA C-acetyltransferase [EC:2.3.1.9] | Carbon metabolism | Dicarboxylate-hydroxybutyrate cycle |
| K14465 |  | succinate semialdehyde reductase (NADPH) [EC:1.1.1.-] | Carbon metabolism | Dicarboxylate-hydroxybutyrate cycle |
| K14534 | *abfD* | 4-hydroxybutyryl-CoA dehydratase / vinylacetyl-CoA-Delta-isomerase [EC:4.2.1.120 5.3.3.3] | Carbon metabolism | Dicarboxylate-hydroxybutyrate cycle |
| K15016 |  | enoyl-CoA hydratase / 3-hydroxyacyl-CoA dehydrogenase [EC:4.2.1.17 1.1.1.35] | Carbon metabolism | Dicarboxylate-hydroxybutyrate cycle |
| K15017 |  | malonyl-CoA/succinyl-CoA reductase (NADPH) [EC:1.2.1.75 1.2.1.76] | Carbon metabolism | Dicarboxylate-hydroxybutyrate cycle |
| K14028 | *mdh1* | methanol dehydrogenase (cytochrome c) subunit 1 [EC:1.1.2.7] | Carbon metabolism | Methane oxidation |
| K16157 | *mmoX* | methane monooxygenase component A alpha chain [EC:1.14.13.25] | Carbon metabolism | Methane oxidation |
| K16161 | *mmoC* | methane monooxygenase component C [EC:1.14.13.25] | Carbon metabolism | Methane oxidation |
| K00194 | *cdhD* | acetyl-CoA decarbonylase/synthase complex subunit delta [EC:2.1.1.245] | Carbon metabolism | Wood-Ljungdahl cycle |
| K00197 | *cdhE* | acetyl-CoA decarbonylase/synthase complex subunit gamma [EC:2.1.1.245] | Carbon metabolism | Wood-Ljungdahl cycle |
| K00198 | *cooS* | anaerobic carbon-monoxide dehydrogenase catalytic subunit [EC:1.2.7.4] | Carbon metabolism | Wood-Ljungdahl cycle |
| K00297 | *metF* | methylenetetrahydrofolate reductase (NADPH) [EC:1.5.1.20] | Carbon metabolism | Wood-Ljungdahl cycle |
| K01491 | *folD* | methylenetetrahydrofolate dehydrogenase (NADP+) / methenyltetrahydrofolate cyclohydrolase [EC:1.5.1.5 3.5.4.9] | Carbon metabolism | Wood-Ljungdahl cycle |
| K01938 | *fhs* | formate--tetrahydrofolate ligase [EC:6.3.4.3] | Carbon metabolism | Wood-Ljungdahl cycle |
| K05299 | *fdhA* | formate dehydrogenase (NADP+) alpha subunit [EC:1.17.1.10] | Carbon metabolism | Wood-Ljungdahl cycle |
| K14138 | *acsB* | acetyl-CoA synthase [EC:2.3.1.169] | Carbon metabolism | Wood-Ljungdahl cycle |
| K15022 | *fdhB* | formate dehydrogenase (NADP+) beta subunit [EC:1.17.1.10] | Carbon metabolism | Wood-Ljungdahl cycle |
| K15023 | *acsE* | 5-methyltetrahydrofolate corrinoid/iron sulfur protein methyltransferase [EC:2.1.1.258] | Carbon metabolism | Wood-Ljungdahl cycle |
| K22015 | *fdhF* | formate dehydrogenase (acceptor) [EC:1.17.99.7] | Carbon metabolism | Wood-Ljungdahl cycle |
| K10944 | *pmoA-amoA* | methane/ammonia monooxygenase subunit A [EC:1.14.18.3 1.14.99.39] | Carbon metabolism/Nitrogen metabolism | Methane oxidation/Nitrification |
| K10945 | *pmoB-amoB* | methane/ammonia monooxygenase subunit B | Carbon metabolism/Nitrogen metabolism | Methane oxidation/Nitrification |
| K10946 | *pmoC-amoC* | methane/ammonia monooxygenase subunit C | Carbon metabolism/Nitrogen metabolism | Methane oxidation/Nitrification |
| K00360 | *nasB* | assimilatory nitrate reductase electron transfer subunit [EC:1.7.99.-] | Nitrogen metabolism | Assimilatory nitrate reduction |
| K00366 | *nirA* | ferredoxin-nitrite reductase [EC:1.7.7.1] | Nitrogen metabolism | Assimilatory nitrate reduction |
| K00367 | *narB* | ferredoxin-nitrate reductase [EC:1.7.7.2] | Nitrogen metabolism | Assimilatory nitrate reduction |
| K00372 | *nasC* | assimilatory nitrate reductase catalytic subunit [EC:1.7.99.-] | Nitrogen metabolism | Assimilatory nitrate reduction |
| K10534 | *NR* | nitrate reductase (NAD(P)H) [EC:1.7.1.1 1.7.1.2 1.7.1.3] | Nitrogen metabolism | Assimilatory nitrate reduction |
| K00368 | *nirK* | nitrite reductase (NO-forming) [EC:1.7.2.1] | Nitrogen metabolism | Denitrification |
| K00376 | *nosZ* | nitrous-oxide reductase [EC:1.7.2.4] | Nitrogen metabolism | Denitrification |
| K02305 | *norC* | nitric oxide reductase subunit C | Nitrogen metabolism | Denitrification |
| K04561 | *norB* | nitric oxide reductase subunit B [EC:1.7.2.5] | Nitrogen metabolism | Denitrification |
| K15864 | *nirS* | nitrite reductase (NO-forming) / hydroxylamine reductase [EC:1.7.2.1 1.7.99.1] | Nitrogen metabolism | Denitrification |
| K00370 | *narG* | nitrate reductase / nitrite oxidoreductase, alpha subunit [EC:1.7.5.1 1.7.99.-] | Nitrogen metabolism | Denitrification/Dissimilatory nitrate reduction |
| K00371 | *narH* | nitrate reductase / nitrite oxidoreductase, beta subunit [EC:1.7.5.1 1.7.99.-] | Nitrogen metabolism | Denitrification/Dissimilatory nitrate reduction |
| K00374 | *narI* | nitrate reductase gamma subunit [EC:1.7.5.1 1.7.99.-] | Nitrogen metabolism | Denitrification/Dissimilatory nitrate reduction |
| K02567 | *napA* | periplasmic nitrate reductase NapA [EC:1.7.99.-] | Nitrogen metabolism | Denitrification/Dissimilatory nitrate reduction |
| K02568 | *napB* | cytochrome c-type protein NapB | Nitrogen metabolism | Denitrification/Dissimilatory nitrate reduction |
| K00362 | *nirB* | nitrite reductase (NADH) large subunit [EC:1.7.1.15] | Nitrogen metabolism | Dissimilatory nitrate reduction |
| K00363 | *nirD* | nitrite reductase (NADH) small subunit [EC:1.7.1.15] | Nitrogen metabolism | Dissimilatory nitrate reduction |
| K03385 | *nrfA* | nitrite reductase (cytochrome c-552) [EC:1.7.2.2] | Nitrogen metabolism | Dissimilatory nitrate reduction |
| K15876 | *nrfH* | cytochrome c nitrite reductase small subunit | Nitrogen metabolism | Dissimilatory nitrate reduction |
| K10535 | *hao* | hydroxylamine dehydrogenase [EC:1.7.2.6] | Nitrogen metabolism | Nitrification |
| K02585 | *nifB* | nitrogen fixation protein NifB | Nitrogen metabolism | Nitrogen fixation |
| K02586 | *nifD* | nitrogenase molybdenum-iron protein alpha chain [EC:1.18.6.1] | Nitrogen metabolism | Nitrogen fixation |
| K02587 | *nifE* | nitrogenase molybdenum-cofactor synthesis protein NifE | Nitrogen metabolism | Nitrogen fixation |
| K02588 | *nifH* | nitrogenase iron protein NifH | Nitrogen metabolism | Nitrogen fixation |
| K02591 | *nifK* | nitrogenase molybdenum-iron protein beta chain [EC:1.18.6.1] | Nitrogen metabolism | Nitrogen fixation |
| K02592 | *nifN* | nitrogenase molybdenum-iron protein NifN | Nitrogen metabolism | Nitrogen fixation |
| K02596 | *nifX* | nitrogen fixation protein NifX | Nitrogen metabolism | Nitrogen fixation |
| K02597 | *nifZ* | nitrogen fixation protein NifZ | Nitrogen metabolism | Nitrogen fixation |
| K02092 | *apcA* | allophycocyanin alpha subunit | Photosynthesis/Pigment | Allophycocyanin |
| K02093 | *apcB* | allophycocyanin beta subunit | Photosynthesis/Pigment | Allophycocyanin |
| K02094 | *apcC* | phycobilisome core linker protein | Photosynthesis/Pigment | Allophycocyanin |
| K02095 | *apcD* | allophycocyanin-B | Photosynthesis/Pigment | Allophycocyanin |
| K02096 | *apcE* | phycobilisome core-membrane linker protein | Photosynthesis/Pigment | Allophycocyanin |
| K02097 | *apcF* | phycobilisome core component | Photosynthesis/Pigment | Allophycocyanin |
| K08942 | *pscC* | photosystem P840 reaction center cytochrome c551 | Photosynthesis/Pigment | Anoxygenic photosystem I |
| K08928 | *pufL* | photosynthetic reaction center L subunit | Photosynthesis/Pigment | Anoxygenic photosystem II |
| K08929 | *pufM* | photosynthetic reaction center M subunit | Photosynthesis/Pigment | Anoxygenic photosystem II |
| K04040 | *chlG* | chlorophyll/bacteriochlorophyll a synthase [EC:2.5.1.62 2.5.1.133] | Photosynthesis/Pigment | Bacterio(chlorophyll) synthesis |
| K10960 | *chlP* | geranylgeranyl diphosphate/geranylgeranyl-bacteriochlorophyllide a reductase [EC:1.3.1.83 1.3.1.111] | Photosynthesis/Pigment | Bacterio(chlorophyll) synthesis |
| K11333 | *bchX* | 3,8-divinyl chlorophyllide a/chlorophyllide a reductase subunit X [EC:1.3.7.14 1.3.7.15] | Photosynthesis/Pigment | Bacteriochlorophyll synthesis |
| K11334 | *bchY* | 3,8-divinyl chlorophyllide a/chlorophyllide a reductase subunit Y [EC:1.3.7.14 1.3.7.15] | Photosynthesis/Pigment | Bacteriochlorophyll synthesis |
| K11335 | *bchZ* | 3,8-divinyl chlorophyllide a/chlorophyllide a reductase subunit Z [EC:1.3.7.14 1.3.7.15] | Photosynthesis/Pigment | Bacteriochlorophyll synthesis |
| K04641 | *bop* | bacteriorhodopsin | Photosynthesis/Pigment | Bacteriorhodopsin |
| K00514 | *ZDS* | zeta-carotene desaturase [EC:1.3.5.6] | Photosynthesis/Pigment | Beta-Carotene biosynthesis |
| K02291 | *crtB* | 15-cis-phytoene synthase [EC:2.5.1.32] | Photosynthesis/Pigment | Beta-Carotene biosynthesis |
| K02293 | *PDS* | 15-cis-phytoene desaturase [EC:1.3.5.5] | Photosynthesis/Pigment | Beta-Carotene biosynthesis |
| K06443 | *lcyB* | lycopene beta-cyclase [EC:5.5.1.19] | Photosynthesis/Pigment | Beta-Carotene biosynthesis |
| K09835 | *crtISO* | prolycopene isomerase [EC:5.2.1.13] | Photosynthesis/Pigment | Beta-Carotene biosynthesis |
| K15744 | *Z-ISO* | zeta-carotene isomerase [EC:5.2.1.12] | Photosynthesis/Pigment | Beta-Carotene biosynthesis |
| K02689 | *psaA* | photosystem I P700 chlorophyll a apoprotein A1 | Photosynthesis/Pigment | Photosystem I |
| K02690 | *psaB* | photosystem I P700 chlorophyll a apoprotein A2 | Photosynthesis/Pigment | Photosystem I |
| K02691 | *psaC* | photosystem I subunit VII | Photosynthesis/Pigment | Photosystem I |
| K02692 | *psaD* | photosystem I subunit II | Photosynthesis/Pigment | Photosystem I |
| K02693 | *psaE* | photosystem I subunit IV | Photosynthesis/Pigment | Photosystem I |
| K02694 | *psaF* | photosystem I subunit III | Photosynthesis/Pigment | Photosystem I |
| K02703 | *psbA* | photosystem II P680 reaction center D1 protein [EC:1.10.3.9] | Photosynthesis/Pigment | Photosystem II |
| K02704 | *psbB* | photosystem II CP47 chlorophyll apoprotein | Photosynthesis/Pigment | Photosystem II |
| K02705 | *psbC* | photosystem II CP43 chlorophyll apoprotein | Photosynthesis/Pigment | Photosystem II |
| K02706 | *psbD* | photosystem II P680 reaction center D2 protein [EC:1.10.3.9] | Photosynthesis/Pigment | Photosystem II |
| K02707 | *psbE* | photosystem II cytochrome b559 subunit alpha | Photosynthesis/Pigment | Photosystem II |
| K02708 | *psbF* | photosystem II cytochrome b559 subunit beta | Photosynthesis/Pigment | Photosystem II |
| K02284 | *cpcA* | phycocyanin alpha chain | Photosynthesis/Pigment | Phycocyanin |
| K02285 | *cpcB* | phycocyanin beta chain | Photosynthesis/Pigment | Phycocyanin |
| K02286 | *cpcC* | phycocyanin-associated rod linker protein | Photosynthesis/Pigment | Phycocyanin |
| K02287 | *cpcD* | phycocyanin-associated, rod | Photosynthesis/Pigment | Phycocyanin |
| K02288 | *cpcE* | phycocyanobilin lyase subunit alpha [EC:4.4.1.32] | Photosynthesis/Pigment | Phycocyanin |
| K02289 | *cpcF* | phycocyanobilin lyase subunit beta [EC:4.4.1.32] | Photosynthesis/Pigment | Phycocyanin |
| K02290 | *cpcG* | phycobilisome rod-core linker protein | Photosynthesis/Pigment | Phycocyanin |
| K05377 | *cpeB* | phycoerythrin beta chain | Photosynthesis/Pigment | Phycoerythrin |
| K05378 | *cpeC* | phycoerythrin-associated linker protein | Photosynthesis/Pigment | Phycoerythrin |
| K05379 | *cpeD* | phycoerythrin-associated linker protein | Photosynthesis/Pigment | Phycoerythrin |
| K05380 | *cpeE* | phycoerythrin-associated linker protein | Photosynthesis/Pigment | Phycoerythrin |
| K05382 | *cpeS* | phycoerythrin-associated linker protein | Photosynthesis/Pigment | Phycoerythrin |
| K05383 | *cpeT* | CpeT protein | Photosynthesis/Pigment | Phycoerythrin |
| K05384 | *cpeU* | bilin biosynthesis protein | Photosynthesis/Pigment | Phycoerythrin |
| K05385 | *cpeY* | bilin biosynthesis protein | Photosynthesis/Pigment | Phycoerythrin |
| K05386 | *cpeZ* | bilin biosynthesis protein | Photosynthesis/Pigment | Phycoerythrin |
| K00380 | *cysJ* | sulfite reductase (NADPH) flavoprotein alpha-component [EC:1.8.1.2] | Sulfur metabolism | Assimilatory sulfate reduction |
| K00381 | *cysI* | sulfite reductase (NADPH) hemoprotein beta-component [EC:1.8.1.2] | Sulfur metabolism | Assimilatory sulfate reduction |
| K00390 | *cysH* | phosphoadenosine phosphosulfate reductase [EC:1.8.4.8 1.8.4.10] | Sulfur metabolism | Assimilatory sulfate reduction |
| K00392 | *sir* | sulfite reductase (ferredoxin) [EC:1.8.7.1] | Sulfur metabolism | Assimilatory sulfate reduction |
| K00860 | *cysC* | adenylylsulfate kinase [EC:2.7.1.25] | Sulfur metabolism | Assimilatory sulfate reduction |
| K00955 | *cysNC* | bifunctional enzyme CysN/CysC [EC:2.7.7.4 2.7.1.25] | Sulfur metabolism | Assimilatory sulfate reduction |
| K00956 | *cysN* | sulfate adenylyltransferase subunit 1 [EC:2.7.7.4] | Sulfur metabolism | Assimilatory sulfate reduction |
| K00957 | *cysD* | sulfate adenylyltransferase subunit 2 [EC:2.7.7.4] | Sulfur metabolism | Assimilatory sulfate reduction |
| K13811 | *PAPSS* | 3'-phosphoadenosine 5'-phosphosulfate synthase [EC:2.7.7.4 2.7.1.25] | Sulfur metabolism | Assimilatory sulfate reduction |
| K00958 | *sat* | sulfate adenylyltransferase [EC:2.7.7.4] | Sulfur metabolism | Assimilatory/dissimilatory sulfate reduction |
| K00394 | *aprA* | adenylylsulfate reductase, subunit A [EC:1.8.99.2] | Sulfur metabolism | Dissimilatory sulfate reduction |
| K00395 | *aprB* | adenylylsulfate reductase, subunit B [EC:1.8.99.2] | Sulfur metabolism | Dissimilatory sulfate reduction |
| K11180 | *dsrA* | dissimilatory sulfite reductase alpha subunit [EC:1.8.99.5] | Sulfur metabolism | Dissimilatory sulfate reduction |
| K11181 | *dsrB* | dissimilatory sulfite reductase beta subunit [EC:1.8.99.5] | Sulfur metabolism | Dissimilatory sulfate reduction |
| K17993 | *hydA* | sulfhydrogenase subunit alpha [EC:1.12.1.3 1.12.1.5] | Sulfur metabolism | Polysulfides reduction |
| K17994 | *hydD* | sulfhydrogenase subunit delta [EC:1.12.1.3 1.12.1.5] | Sulfur metabolism | Polysulfides reduction |
| K17995 | *hydG* | sulfhydrogenase subunit gamma (sulfur reductase) [EC:1.12.98.4] | Sulfur metabolism | Polysulfides reduction |
| K17996 | *hydB* | sulfhydrogenase subunit beta (sulfur reductase) [EC:1.12.98.4] | Sulfur metabolism | Polysulfides reduction |
| K17218 | *sqr* | sulfide:quinone oxidoreductase [EC:1.8.5.4] | Sulfur metabolism | Sulfides oxidation |
| K17229 | *fccB* | sulfide dehydrogenase [flavocytochrome c] flavoprotein chain [EC:1.8.2.3] | Sulfur metabolism | Sulfides oxidation |
| K17230 | *fccA* | cytochrome subunit of sulfide dehydrogenase | Sulfur metabolism | Sulfides oxidation |
| K00387 | *SUOX* | sulfite oxidase [EC:1.8.3.1] | Sulfur metabolism | Sulfite oxidation |
| K05301 | *sorA* | sulfite dehydrogenase [EC:1.8.2.1] | Sulfur metabolism | Sulfite oxidation |
| K21307 | *soeA* | sulfite dehydrogenase (quinone) subunit SoeA [EC:1.8.5.6] | Sulfur metabolism | Sulfite oxidation |
| K21308 | *soeB* | sulfite dehydrogenase (quinone) subunit SoeB | Sulfur metabolism | Sulfite oxidation |
| K21309 | *soeC* | sulfite dehydrogenase (quinone) subunit SoeC | Sulfur metabolism | Sulfite oxidation |
| K16950 | *asrA* | anaerobic sulfite reductase subunit A | Sulfur metabolism | Sulfite reduction |
| K16951 | *asrB* | anaerobic sulfite reductase subunit B | Sulfur metabolism | Sulfite reduction |
| K08357 | *ttrA* | tetrathionate reductase subunit A | Sulfur metabolism | Tetrathionate reduction |
| K08358 | *ttrB* | tetrathionate reductase subunit B | Sulfur metabolism | Tetrathionate reduction |
| K08359 | *ttrC* | tetrathionate reductase subunit C | Sulfur metabolism | Tetrathionate reduction |
| K01011 | *TST* | thiosulfate/3-mercaptopyruvate sulfurtransferase [EC:2.8.1.1 2.8.1.2] | Sulfur metabolism | Thiosulfate oxidation |
| K16936 | *doxA* | thiosulfate dehydrogenase [quinone] small subunit [EC:1.8.5.2] | Sulfur metabolism | Thiosulfate oxidation |
| K16937 | *doxD* | thiosulfate dehydrogenase [quinone] large subunit [EC:1.8.5.2] | Sulfur metabolism | Thiosulfate oxidation |
| K17222 | *soxA* | L-cysteine S-thiosulfotransferase [EC:2.8.5.2] | Sulfur metabolism | Thiosulfate oxidation |
| K17223 | *soxX* | L-cysteine S-thiosulfotransferase [EC:2.8.5.2] | Sulfur metabolism | Thiosulfate oxidation |
| K17224 | *soxB* | S-sulfosulfanyl-L-cysteine sulfohydrolase [EC:3.1.6.20] | Sulfur metabolism | Thiosulfate oxidation |
| K17225 | *soxC* | sulfane dehydrogenase subunit SoxC | Sulfur metabolism | Thiosulfate oxidation |
| K17226 | *soxY* | sulfur-oxidizing protein SoxY | Sulfur metabolism | Thiosulfate oxidation |
| K17227 | *soxZ* | sulfur-oxidizing protein SoxZ | Sulfur metabolism | Thiosulfate oxidation |
| K08352 | *phsA* | thiosulfate reductase / polysulfide reductase chain A [EC:1.8.5.5] | Sulfur metabolism | Thiosulfate reduction |
| K08354 | *phsC* | thiosulfate reductase cytochrome b subunit | Sulfur metabolism | Thiosulfate reduction |

**Table S2.** Differential abundance analysis results for the KOs listed in Table S1 that are significantly (*p*≤0.01) differentially abundant along the river between the shallow and the deeper sites (*RSh* vs. *R*).

| KO | Gene | Log2FoldChange *RSh* vs *R* | Adjusted *p*-values |
| --- | --- | --- | --- |
| K00024 | *mdh* | -0.3953 | *p*<0.001 |
| K00031 | *IDH1* | -0.2304 | *p*<0.001 |
| K00134 | *GAPDH* | -0.1782 | *p*<0.001 |
| K00150 | *gap2* | 2.7141 | *p*<0.001 |
| K00169 | *porA* | 1.7353 | *p*<0.001 |
| K00170 | *porB* | 1.5919 | *p*<0.001 |
| K00174 | *korA* | 0.4409 | *p*<0.001 |
| K00175 | *korB* | 0.3340 | *p*=0.002 |
| K00239 | *sdhA* | -0.3958 | *p*<0.001 |
| K00240 | *sdhB* | -0.4460 | *p*<0.001 |
| K00244 | *frdA* | -0.5246 | *p*<0.001 |
| K00297 | *metF* | -0.3035 | *p*<0.001 |
| K00366 | *nirA* | 1.2198 | *p*<0.001 |
| K00367 | *narB* | 2.1982 | *p*<0.001 |
| K00368 | *nirK* | -0.3058 | *p*<0.001 |
| K00370 | *narG* | 0.6184 | *p*<0.001 |
| K00376 | *nosZ* | 1.3644 | *p*=0.005 |
| K00392 | *sir* | 0.6224 | *p*<0.001 |
| K00615 | *E2.2.1.1* | -0.3098 | *p*<0.001 |
| K00927 | *PGK* | 1.5971 | *p*<0.001 |
| K00958 | *sat* | -0.2959 | *p*<0.001 |
| K01011 | *TST* | -0.4436 | *p*<0.001 |
| K01491 | *folD* | -0.6253 | *p*<0.001 |
| K01595 | *ppc* | -0.4631 | *p*<0.001 |
| K01624 | *FBA* | 0.4052 | *p*<0.001 |
| K01676 | *E4.2.1.2A* | -0.3685 | *p*<0.001 |
| K01679 | *E4.2.1.2B* | -0.4683 | *p*<0.001 |
| K01681 | *ACO* | -0.3923 | *p*<0.001 |
| K01682 | *acnB* | 0.4035 | *p*<0.001 |
| K01808 | *rpiB* | -0.4309 | *p*<0.001 |
| K01848 | *E5.4.99.2A* | -0.4209 | *p*<0.001 |
| K01902 | *sucD* | 0.8570 | *p*<0.001 |
| K01903 | *sucC* | 2.0990 | *p*<0.001 |
| K01958 | *PC* | 2.9902 | *p*<0.001 |
| K02092 | *apcA* | 2.0309 | *p*=0.002 |
| K02093 | *apcB* | 2.1352 | *p*<0.001 |
| K02094 | *apcC* | 2.5989 | *p*<0.001 |
| K02095 | *apcD* | 2.0152 | *p*=0.004 |
| K02096 | *apcE* | 2.2895 | *p*<0.001 |
| K02097 | *apcF* | 2.1269 | *p*=0.003 |
| K02284 | *cpcA* | 2.2652 | *p*<0.001 |
| KO | Gene | Log2FoldChange  *RSh* vs *R* | Adjusted *p*-values |
| K02285 | *cpcB* | -0.3991 | *p*<0.001 |
| K02288 | *cpcE* | 1.4133 | *p*=0.001 |
| K02290 | *cpcG* | 2.1492 | *p*<0.001 |
| K02291 | *crtB* | 2.1425 | *p*<0.001 |
| K02293 | *PDS* | 2.2149 | *p*<0.001 |
| K02689 | *psaA* | 2.8526 | *p*<0.001 |
| K02690 | *psaB* | 1.7441 | *p*<0.001 |
| K02691 | *psaC* | 3.1613 | *p*=0.005 |
| K02692 | *psaD* | 2.4935 | *p*=0.002 |
| K02703 | *psbA* | 1.2981 | *p*<0.001 |
| K02704 | *psbB* | 2.9038 | *p*<0.001 |
| K02705 | *psbC* | 3.5530 | *p*<0.001 |
| K02706 | *psbD* | 1.5155 | *p*<0.001 |
| K02707 | *psbE* | 2.0793 | *p*=0.003 |
| K03385 | *nrfA* | -0.7995 | *p*<0.001 |
| K03737 | *por* | 2.4010 | *p*<0.001 |
| K04561 | *norB* | 1.2955 | *p*<0.001 |
| K05301 | *sorA* | -0.7762 | *p*<0.001 |
| K05378 | *cpeC* | -0.7753 | *p*<0.001 |
| K08928 | *pufL* | -0.7488 | *p*=0.002 |
| K08929 | *pufM* | 0.6458 | *p*<0.001 |
| K09709 | *meh* | 3.3103 | *p*<0.001 |
| K09835 | *crtISO* | -1.0214 | *p*<0.001 |
| K11334 | *bchY* | -0.8122 | *p*<0.001 |
| K11335 | *bchZ* | -0.4967 | *p*<0.001 |
| K14028 | *mdh1* | -0.7280 | *p*<0.001 |
| K16937 | *doxD* | -1.0214 | *p*<0.001 |
| K17218 | *sqr* | 0.6255 | *p*<0.001 |
| K17222 | *soxA* | -0.7267 | *p*<0.001 |
| K17223 | *soxX* | -0.8122 | *p*<0.001 |
| K17224 | *soxB* | -0.4967 | *p*<0.001 |
| K17225 | *soxC* | -0.3518 | *p*=0.001 |
| K17226 | *soxY* | -0.7280 | *p*<0.001 |

**Table S3.** Differential abundance analysis results for the KOs listed in Table S1 that are significantly (*p*≤0.01) differentially abundant in the vertical plume profile between surface water and brackish water at 4 m depth (*PS* vs. *P4M*).

| KO | Gene | Log2FoldChange  *PS* vs *P4M* | Adjusted *p*-values |
| --- | --- | --- | --- |
| K00150 | *gap2* | -3.0733 | *p*<0.001 |
| K00394 | *aprA* | -4.6521 | *p*<0.001 |
| K00395 | *aprB* | -4.6041 | *p*<0.001 |
| K00958 | *sat* | -2.4451 | *p*<0.001 |
| K01623 | *ALDO* | -1.1295 | *p*=0.001 |
| K01959 | *pycA* | -1.7546 | *p*=0.002 |
| K02690 | *psaB* | -2.0400 | *p*=0.002 |
| K02704 | *psbB* | -2.7004 | *p*<0.001 |
| K05299 | *fdhA* | -3.7424 | *p*<0.001 |
| K08352 | *phsA* | -1.3395 | *p*<0.001 |
| K08358 | *ttrB* | -3.0613 | *p*<0.001 |
| K10945 | *pmoB-amoB* | -3.7843 | *p*<0.001 |
| K10946 | *pmoC-amoC* | -3.8718 | *p*<0.001 |
| K11532 | *glpX-SEBP* | -1.2838 | *p*<0.001 |
| K14449 | *mch* | -1.4795 | *p*<0.001 |
| K15016 |  | -3.9818 | *p*<0.001 |
